# Tender Nodules of the Foot

**Published:** 2018-01-01

**Authors:** Carol Guarnieri

**Affiliations:** HonorHealth Research Institute, Scottsdale, Arizona

**HISTORY**

The patient is a 56-year-old woman who suffered a significant left foot injury in 2010 when her left foot was crushed in a car accident. Due to her injury, she favored bearing weight on her right foot. In late 2015, she started having pain along the lateral aspect of her right foot. She thought it was due to the increased pressure she was applying to her right foot and did not seek medical attention at that time.

However, in July 2016, she felt a nodule around her lateral right ankle. She saw her primary care physician (PCP) for an evaluation. Her PCP initially thought the nodule was a ganglion cyst and tried to aspirate it on two occasions, but the aspirations were unsuccessful. The PCP was concerned that the nodule was a malignant lesion; therefore, the patient was subsequently referred to a surgical orthopedic oncologist who arranged for a magnetic resonance imaging (MRI) scan of her foot.

**Figure 1:**
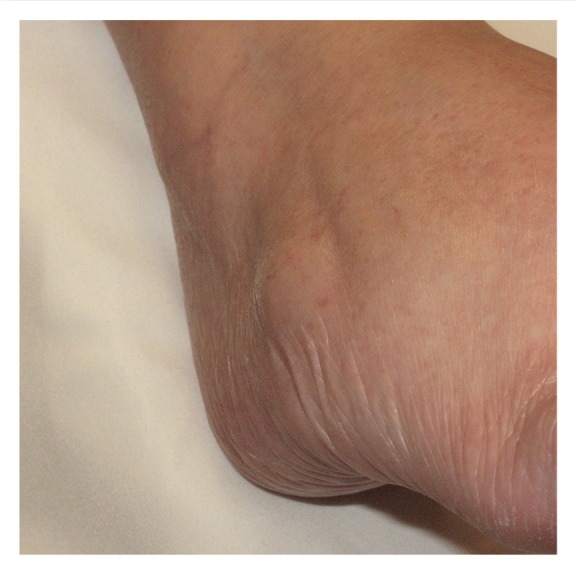
Figure 1

**Figure 2:**
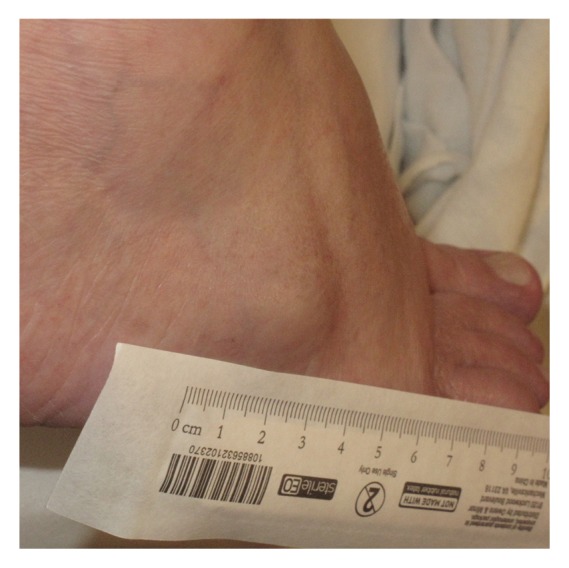
Figure 2

The MRI demonstrated an infiltrative process involving the synovium of the tarsal bones with a lobulated lesion measuring 2.8 × 1.5 × 2 cm. There were similar additional nodular lesions along the plantar surface of the tarsal bones with the MRI documenting a total of five separate lesions. It also demonstrated bony erosion of the posterior aspect of the cuboid and lateral cuneiform bones.

Upon physical examination, multiple tender nodules were palpated along the outer aspect of the right foot without erythema or edema. She was unable to heel walk or toe walk due to pain and was unable to ambulate long distances due to constant pain, using a cane for support. She took an occasional hydrocodone-acetaminophen 5-325 mg per tablet for pain with good relief.

Her medical history included hypertension which was controlled by medication. Surgeries include a resection of an adrenal mass in 2009 for Conn’s syndrome and a hysterectomy in 2014 for nonmalignant reasons.

**Figure 3:**
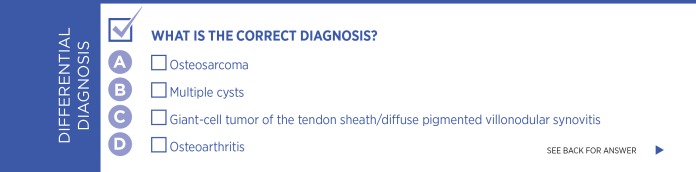
What is the correct diagnosis?

## CORRECT ANSWER: C

**Giant-cell tumor of the tendon sheath/diffuse pigmented villonodular synovitis.** An ultrasound-guided biopsy was performed. Pathology showed histologic features of the biopsy to be compatible with localized giant-cell tumor of the tendon sheath, also known as diffuse pigmented villonodular synovitis (PVNS).

Pigmented villonodular synovitis, also known as tenosynovial giant-cell tumor, is a rare synovial proliferative disease with an incidence of 1.8 cases per 1 million people (Chen et al., 2015). Due to its rare incidence, the disease remains a diagnostic challenge. Pigmented villonodular synovitis is characterized by thickened and overgrown synovium. The synovium is the thin layer of tissue that lines the joints and tendons. As a result of this overgrowth, PVNS proliferates and invades into the articular cartilage. The etiology of PVNS is unclear. Although PVNS is considered to be a benign disease, malignant cases have been reported. Nonetheless, this benign proliferation can produce destruction of the joint, pain, swelling, and considerable functional deterioration (Ravi, Wang, & Lewis, 2011). Pigmented villonodular synovitis typically involves only one joint with 80% of cases affecting the knee, but can alternatively affect the hip, ankle, shoulder, foot, or elbow. Patients usually present with a localized, painful, slow-growing mass. Pigmented villonodular synovitis generally occurs in patients between the ages of 20 to 45 years.

There are two forms of PVNS: localized and diffuse. Localized tumors usually involve one joint and are generally indolent, whereas diffuse tumors are locally aggressive, more destructive, and more difficult to treat.

Surgical removal of all diseased synovial lining remains the standard of care for disease eradication. However, surgery in diffuse tumors often fails to obtain definitive disease control (Ravi et al., 2011). It is not uncommon for patients with diffuse PVNS to have multiple recurrences and require several surgical procedures that result in functional impairments. Unfortunately, it is almost impossible to excise the mass in its entirety via synovectomy without causing joint damage, as there is no boundary that exists between the lesions and normal tissue (Stacchiotti et al., 2013). Recurrence rates with partial synovectomy are as high as 56% within 2 years (Startzman, Collins, & Carreira, 2016). Pigmented villonodular synovitis within the hip carries a worse prognosis than that found in other joints, likely due to the large space within the hip and an increased time to diagnosis (Byrd, Jones, Maiers, 2013).

West and colleagues (2006) identified a colony-stimulating factor 1 receptor (*CSF1R*) gene, which encodes for the ligand of CSF1R at the chromosome 1p13 breakpoint and found it to be translocated in 63% to 77% of patients with PVNS. CSF1R ligand is overexpressed in PVNS due to a specific t(1;2) translocation, which fuses the *CSF1* gene to the collagen type VI alpha-3 promoter. This recurrent translocation leads to CSF1 overexpression, which plays an active role in the pathogenesis of these tumors (Cupp et al., 2007). The results of the phase I trial show that blocking the CSF1 pathway in tenosynovial giant-cell tumors with an appropriately designed therapy can induce significant regressions in tumor volume (Tap et al., 2015).

Imatinib mesylate (Gleevec), a tyrosine kinase inhibitor that is used in chronic myeloid leukemia and gastrointestinal stromal cell tumor, has shown a clinical effect on PVNS. To date, imatinib mesylate is not approved for use in PVNS, but there is strong evidence to support its use.

Imatinib mesylate’s antitumor effect is thought to be the blockade of CSF1R. In a multi-institutional retrospective study of 27 patients, imatinib mesylate showed a Response Evaluation Criteria in Solid Tumors (RECIST) partial response in 19% of patients and stable disease in 74% of patients (Cassier et al., 2012).

## EXPLANATION OF INCORRECT ANSWERS

**Osteosarcoma.** This can be included in the differential diagnosis. Our patient is older than the average age of patients with osteosarcoma (the average age is between 10–30 years old; American Cancer Society, 2017). In addition, osteosarcoma usually does not present with a foot lesion. It is most commonly seen in the femur, tibia, and humerus.

**Multiple cysts.** On two separate occasions, aspirations were attempted and were unsuccessful. It wasn’t until the MRI was done that it was revealed to be a solid mass, not a cystic lesion.

**Osteoarthritis.** Osteoarthritis usually affects joints in the hands, knees, hips, and spine. It usually presents with joint pain or stiffness that is most noticeable when waking up in the morning or after a period of inactivity. A unilateral palpable mass in the foot is not a common presentation for osteoarthritis.

## CURRENT TREATMENT PLAN

The patient was referred to our clinic after the orthopedic surgeon received the results of her biopsy and her case was presented at the sarcoma tumor board meeting. The orthopedic surgeon had a discussion with the patient on the surgical treatment option that would involve amputation of her right foot. She was offered the opportunity to enroll in a clinical trial of a CSF1R inhibitor, which has shown very encouraging results in early studies of this oral medication or to receive the standard of care and start on imatinib mesylate. The patient chose to enroll in the clinical trial and completed two cycles of the study drug. She is tolerating the medication well with minimal side effects. She is experiencing no pain with ambulation and no longer needs the assistance of a cane. She will continue in the clinical trial until she shows progression of disease by RECIST criteria.

A clinical trial is an excellent option for rare cancers with agents to improve upon the treatment standard or where there is often no treatment available. Advanced practitioners are in a prime position to educate and refer patients for clinical trials.
